# Long-Term Outcome of Neoadjuvant Endocrine Therapy with Aromatase Inhibitors in Elderly Women with Hormone Receptor-Positive Breast Cancer

**DOI:** 10.1245/s10434-014-3535-7

**Published:** 2014-02-13

**Authors:** Antonino Grassadonia, Marta Di Nicola, Simona Grossi, Paolo Noccioli, Saveria Tavoletta, Roberto Politi, Domenico Angelucci, Camilla Marinelli, Marinella Zilli, Giampiero Ausili Cefaro, Nicola Tinari, Michele De Tursi, Laura Iezzi, Pasquale Cioffi, Stefano Iacobelli, Clara Natoli, Ettore Cianchetti

**Affiliations:** 1 Medical Oncology Unit, Department of Experimental and Clinical Sciences, University ‘G. d’Annunzio’, Chieti, Italy; 2 Laboratory of Biostatistics, Department of Experimental and Clinical Science, University ‘G. d’Annunzio’, Chieti, Italy; 3 Division of Surgical Senology, ‘G. Bernabeo’ Hospital, Ortona, CH Italy; 4 Division of Pathology, ‘SS. Annunziata’ Hospital, Chieti, Italy; 5 Oncology Department, ‘SS. Annunziata’ Hospital, Chieti, Italy; 6 Radiation Oncology Department, University ‘G. d’Annunzio’, Chieti, Italy; 7 Hospital Pharmacy, ‘SS. Annunziata’ Hospital, Chieti, Italy; 8 Division of Surgical Senology, Department of Experimental and Clinical Sciences, University ‘G. d’Annunzio’, Chieti, Italy

## Abstract

**Background:**

Aromatase inhibitors (AIs) are more effective than tamoxifen as neoadjuvant endocrine therapy (NET) for hormone receptor (HR)-positive breast cancer. Here we report the surgical and long-term outcome of elderly postmenopausal patients with locally advanced, HR-positive breast cancer treated with preoperative AIs.

**Methods:**

Between January 2003 and December 2012, 144 postmenopausal patients inoperable with breast conservative surgery (BCS) received letrozole, anastrozole, or exemestane as NET. Patients underwent breast surgery and received adjuvant AIs. Adjuvant systemic therapy, chemotherapy and/or trastuzumab, and adjuvant radiotherapy were administered as appropriate, but limited to high-risk patients with few or no comorbidities.

**Results:**

After a median follow-up of 49 months, 4 (3.0 %) patients had local relapse, 18 (12.5 %) had distant metastases, and 24 (17.0 %) died. BCS was performed in 121 (84.0 %) patients. A tumor size <3 cm and human epidermal growth factor receptor 2 (HER2) negativity were predictors of BCS. The achievement of BCS and grade G1 were significantly associated with longer disease-free survival (DFS) (*p* = 0.009 and *p* = 0.01, respectively) and overall survival (*p* = 0.002 and *p* = 0.005, respectively). Residual tumor ≤2 cm (yT0–yT1) in the longest diameter after NET was also statistically associated with longer DFS (*p* = 0.005).

**Conclusions:**

The results of this retrospective study indicate that elderly breast cancer patients with a tumor size <3 cm at diagnosis and HER2 negativity have a higher probability of achieving BCS after NET. Moreover, patients treated with BCS and with grade G1 tumor have a reduced risk of recurrence and death in the long-term follow-up.

Neoadjuvant endocrine therapy (NET) has been historically administered to patients with locally advanced breast cancer and unfit for chemotherapy because of age and/or comorbidities.^1^,^2^ New perspectives on the use of NET in healthy postmenopausal women have recently emerged on the basis of studies showing that patients with hormone receptor (HR)-positive breast cancer hardly achieve pathologic complete response (pCR) after neoadjuvant chemotherapy, thus suggesting that HR-positive tumors are quite resistant to this therapeutic approach.^3^–^10^ Moreover, patients who do not achieve a pCR to neoadjuvant chemotherapy maintain a good prognosis even in the presence of residual disease.^11^,^12^ The good outcome of these patients is largely dependent on the efficacy of postoperative endocrine therapy, especially when based on the third-generation aromatase inhibitors (AIs) letrozole, anastrozole, and exemestane. In postmenopausal women, adjuvant AIs have been shown to be superior to tamoxifen in terms of disease-free survival (DFS) and overall survival (OS).^13^–^15^ Similarly, in the neoadjuvant treatment of postmenopausal women with breast cancer, AIs allow higher rates of objective responses and breast conservative surgery (BCS) to be achieved than tamoxifen.^16^–^19^

Few studies have assessed the impact of response to neoadjuvant AI on the OS of patients with HR-positive breast cancer. This study was conducted to evaluate the long-term outcome of elderly postmenopausal women with locally advanced HR-positive breast cancer treated with neoadjuvant AIs in our institution.

## Patients and Methods

### Patients

The study population was identified by a systematic review of the chart of all women with locally advanced breast cancer, candidates to mastectomy, and consecutively treated with NET—letrozole, anastrozole, or exemestane—between January 2003 and December 2012. All patients had a diagnosis of HR-positive invasive breast cancer as established by tru-cut biopsy of the primary tumor and immunohistochemistry (IHC), and were postmenopausal. The study has been approved by our institutional Ethics Committee.

### Treatments

Mastectomy or BCS were performed after NET as indicated by the surgeon. Axillary lymph node dissection or sentinel node biopsy were performed according to surgeon judgment. After surgery, AIs were continued as adjuvant treatment in all patients. Adjuvant chemotherapy was administered to high-risk, non-responsive patients with few or no comorbidities. Adjuvant trastuzumab was also considered for patients with human epidermal growth factor receptor 2 (HER2)-positive tumor. Adjuvant breast radiotherapy was delivered to patients who underwent BCS and to patients treated with mastectomy and stage cT3, cN2 or cN3 at diagnosis or stage pN2 after surgery. However, in patients unfit for age or comorbidities, radiotherapy was not administered.^20^

### Clinical Evaluation

The clinical response to NET was evaluated by measuring the largest diameter of the tumor by caliper at baseline, every month and just before surgery. Mammography and breast ultrasound were also performed, but data were not available for all patients. The Response Evaluation Criteria in Solid Tumors (RECIST) were utilized to define clinical responses: complete response (CR), partial response (PR), stable disease (SD), and progressive disease (PD).^21^

### Pathological Assessment

Tru-cut biopsies and surgical specimens were both processed to determine morphological and molecular features. Histological type and grade of carcinoma were assessed on hematoxylin and eosin-stained tumor sections. The tumor grade was scored according to the Elston–Ellis classification. The expression of estrogen (ER), progesterone receptors (PR), HER2, and Ki-67 were evaluated by IHC. Cut-off positivity for HR was fixed at 10 % of tumor cells stained for ER and/or PR.^22^ HER2 status was assessed by HercepTest (Dako Italia, Milan, Italy). Tumors with a score of 3+ by IHC, or gene amplification by fluorescence or chromogenic in situ hybridization (FISH or CISH), were considered as HER2 positive. IHC detection of Ki-67 was performed using the MIB-1 antibody.

pCR was defined as the absence of invasive cancer within the breast (ypT0/is) and lymph node (ypN0), after extensive sampling, i.e. at least ten sections, 2–4 μm in thickness, from three different regions of the initial tumor site, as proposed by Kuerer et al.^12^ Pathological stages were categorized according to the American Joint Committee on Cancer Staging Manual, 7th edition.

### Statistical Analysis

Logistic regression was applied to identify variables predictive of BCS. The results of the model were expressed as odds ratio (OR) and relative 95 % confidence interval (CI). Multivariate logistic regression was applied to evaluate the adjusted ORs. The Kaplan–Meier method was used to calculate the 5-year rates of DFS and OS. OS was defined as the time between surgery and death or last follow-up visit, and DFS as the time between surgery and the first verified event. Differences between curves were evaluated by the log-rank test. To identify independent prognostic factors with significant impact on DFS and OS, multivariate analyses were performed using the Cox proportional hazards model. Calculating the exponential of the regression coefficients from the Cox model provided an estimate of the hazard ratio (HR) and the 95 % CI. Stability of models was guaranteed by backward fitting procedure. A *p* value of 0.05 or less was considered statistically significant. All statistical analysis was performed using SPSS^®^ software 11.0 (SPSS Inc, Chicago, IL, USA).

## Results

### Patient and Tumor Characteristics at Baseline

Overall, 144 patients were identified and included in the study. All patients were treated with third-generation AIs: 56 (38.9 %) patients received letrozole, 83 (57.6 %) exemestane, and 5 (3.5 %) anastrozole. Patients’ characteristics are illustrated in Table 1. Mean age was 76.4 years (±8.2 years), with 131 (90.3 %) patients being older than 65 years. More than half of the study population had a tumor size >3 cm in the largest diameter, and the most frequent cancer histotype was invasive ductal carcinoma. Tumor grade was G1 in 95 (66 %) patients, and Ki-67 was ≤14 % in 88 (61.1 %) patients. Only 13 (9.0 %) patients had HER2-positive tumor. The mean duration of NET was 5.7 months (±1.5 months).

**Table 1:** Pre-treatment clinical characteristics of patients

Variable	
Mean age at surgery, years (mean ± SD)	76.4 ± 8.2
Age at surgery (years) [*n* (%)]	
≤65	14 (9.7)
>65	131 (90.3)
Clinical T (cm) [*n* (%)]	
≤3	66 (45.8)
>3	78 (54.2)
Histologic type [*n* (%)]	
Ductal	137 (95.1)
Lobular	5 (3.5)
Others	2 (1.4)
Tumor grade [*n* (%)]	
G1	95 (66.0)
G2	38 (26.4)
G3	4 (2.8)
Unknown	7 (4.8)
Molecular subtype [*n* (%)]	
HER*-*2 negative	131 (91.0)
HER*-*2 positive	13 (9.0)
Ki-67 (%) [*n* (%)]	
≤14	88 (61.1)
>14	40 (27.8)
Unknown	16 (11.1)
Duration of NET, months (mean ± SD)	5.7 ± 1.5

*BCS* breast conservative surgery, *NET* neoadjuvant endocrine therapy, *HER-2* human epidermal growth factor receptor 2

### Clinical Response and Surgery Outcome

Of 135 patients evaluable for clinical response, CR was obtained in 13 (9.6 %), PR in 104 (77.0 %), SD in 16 (11.8 %), and PD in 2 (1.5 %). The type of hormonal agent used did not significantly influence clinical response (data not shown). With the exception of four patients with PR who required mastectomy, all patients reporting objective response (CR + PR) received BCS. Axillary lymph node dissection was performed in 97 (67.4 %) patients, including nine with nodal involvement at sentinel node biopsy at surgery.

After NET, BCS was performed in 121 (84 %) patients and mastectomy in 23 (16 %) patients. The probability of achieving BCS according to the clinicopathologic characteristics of patients is shown in Table 2. At univariate analyses, factors predictive for BCS were cT ≤ 3 cm (*p* = 0.031), HER-2 negativity (*p* = 0.002), and grade G1 (*p* = 0.02). At multivariate analyses, only a small tumor size, i.e. cT ≤ 3 cm at diagnosis (*p* = 0.017) and HER-2 negativity (*p* = 0.05) remained significant predictors of BCS.

**Table 2:** Univariate and multivariate analysis of variables predictive of BCS surgery

Variable	Univariate		Multivariate	
	Odds ratio (95 % CI)	*p* Value	Odds ratio (95 % CI)	*p* Value
Age at surgery (years)				
>65	1.00		1.00	
≤65	2.42 (0.30–19.59)	0.407	2.92 (0.27–21.37)	0.376
Clinical T (cm)				
>3	1.00		1.00	
≤3	2.79 (1.03–7.55)	**0.044**	5.48 (1.35–20.20)	**0.017**
Molecular subtype				
HER-2 positive	1.00		1.00	
HER-2 negative	5.75 (1.72–19.15)	**0.004**	4.93 (1.09–19.85)	**0.050**
Grade				
G2–G3	1.00		1.00	
G1	3.01 (1.17–7.80)	**0.023**	2.35 (0.68–8.12)	0.175
Ki-67 (%)				
>14	1.00		1.00	
≤14	2.03 (0.77–5.39)	0.154	2.37 (0.54–10.27)	0.250
Duration of NET	1.05 (0.77–1.43)	0.753	0.99 (0.66–1.50)	0.942

Bold values indicate significant *p* values

‘Unknown’ were not included in the analysis

*BCS* breast conservative surgery, *NET* neoadjuvant endocrine therapy, *CI* confidence interval, *HER-2* human epidermal growth factor receptor 2

### Pathological Response and Adjuvant Therapy

A pCR (ypT0/is; ypN0) was obtained in only two patients, and absence of cancer in the breast but not in the nodes (ypT0/is; ypN1) in three patients. After surgery, adjuvant treatment with AIs was continued in all patients, but in 22 patients it was preceded by adjuvant chemotherapy. A total of 125 (87 %) patients maintained the same AI in the adjuvant setting. Patients with non-responsive tumor were switched to another non-cross-resistant agent: 14 (10 %) from letrozole to exemestane, and 5 (3 %) from exemestane to letrozole. Of 13 patients with HER2-positive tumors, 7 received trastuzumab, in 4 cases in combination with chemotherapy.

Adjuvant radiotherapy was delivered to 77 (64 %) of 121 patients who underwent BCS, and to 5 (22 %) of 23 patients who underwent mastectomy. Radiotherapy was not delivered to 44 (36 %) patients with BCS and to 9 (64 %) of 14 patients with mastectomy.

### Long-Term Outcome

**Fig. 1:**
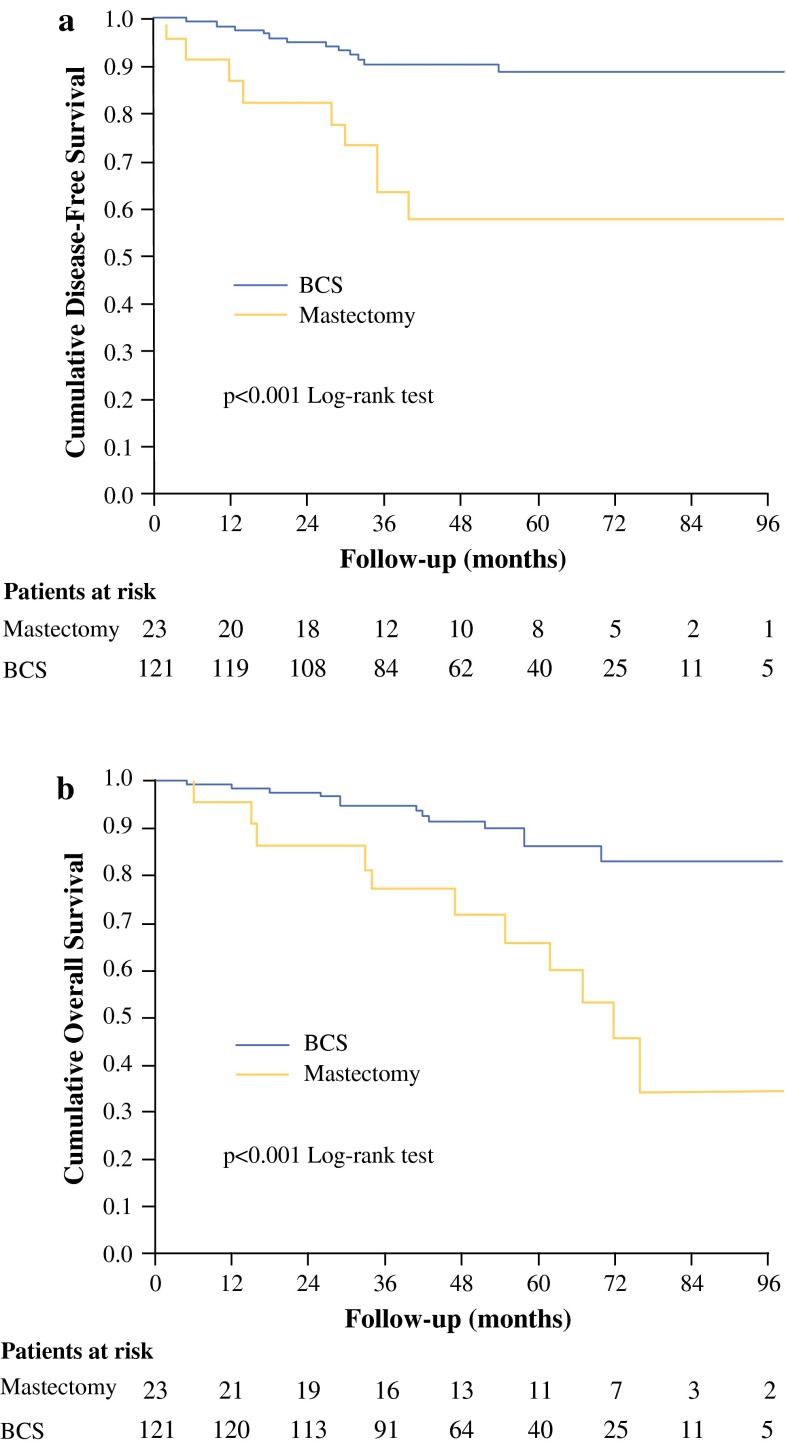
Cumulative disease-free survival **a** and overall survival **b** stratified by type of surgery. *BCS* breast conservative surgery

After a median follow-up of 49 months (range 3–119 months), 4 (3 %) patients had a local relapse, 18 (12.5 %) had distant metastases, and 24 (17 %) died. Results of univariate analysis of factors associated with DFS and OS are shown in Table 3. In particular, HER-2-negative tumor (*p* = 0.08), grade G1 (*p* < 0.001), achievement of BCS (*p* = 0.001), Ki-67 ≤ 14 % (*p* = 0.002), residual tumor ≤2 cm (*p* = 0.007), number of metastatic nodes ≤3 (*p* = 0.001), administration of adjuvant chemotherapy (*p* = 0.007), and stage I disease after surgery (*p* = 0.004) were associated with a better DFS. Only BCS and grade G1 (*p* = 0.001) were predictors of a better OS. Figure 1 refers to Kaplan–Meier analysis of DFS (Fig. 1a) and OS (Fig. 1b) according to type of surgery. At 5 years of follow-up, the estimated cumulative DFS rate was 88.6 % for BCS and 57.5 % for mastectomy, while the estimated cumulative OS rate was 86.1 % for BCS and 65.8 % for mastectomy.

**Table 3:** Univariate analysis of factors predictive of 5-year disease-free survival and overall survival

Variable	*n*	Disease-free survival			Overall survival		
		5-year (%)^a^	HR (95 % CI)	*p* Value	5-year (%)^a^	HR (95 % CI)	*p* Value
Age at surgery (years)							
≤65	14	83.1	1.00		92.3	1.00	
>65	131	83.5	1.02 (0.24–4.37)	0.981	81.7	1.12 (0.26–4.82)	0.882
Molecular subtype							
HER-2 negative	131	86.4	1.00		83.7	1.00	
HER-2 positive	13	47.5	3.93 (1.44–10.75)	**0.008**	70.3	1.92 (0.65–5.64)	0.235
Grade (basal)							
G1	95	94.3	1.00		91.8	1.00	
G2–G3	42	57.3	8.57 (3.14–23.42)	**<0.001**	62.6	4.35 (1.84–10.27)	**0.001**
Type of surgery							
BCS	121	88.6	1.00		86.1	1.00	
Mastectomy	23	57.5	4.46 (1.88–10.59)	**0.001**	65.8	4.15 (1.85–9.29)	**0.001**
Ki67 (basal; %)							
≤14	88	88.9	1.00		81.7	1.00	
>14	40	64.6	4.09 (1.67–10.04)	**0.002**	76.1	1.53 (0.66–3.55)	0.317
T stage (after surgery)							
yT0–yT1	66	94.5	1.00		85.6	1.00	
yT2–yT3	78	74.5	5.42 (1.60–18.41)	**0.007**	79.7	2.36 (0.94–5.95)	0.069
No. of metastatic nodes							
None	85	91.1	1.00		83.6	1.00	
≤3	34	86.7	1.34 (0.39–4.58)	0.640	88.1	1.48 (0.13–1.81)	0.259
>3	25	55.9	5.56 (2.12–11.62)	**0.001**	68.8	2.22 (0.93–5.31)	0.073
Adjuvant RT							
No	62	86.7	1.00		80.6	1.00	
Yes	82	80.9	0.64 (0.26–1.60)	0.344	85.2	1.38 (0.62–3.09)	0.425
Adjuvant chemotherapy							
No	122	88.3	1.00		84.1	1.00	
Yes	22	61.5	3.36 (1.39–8.11)	**0.007**	75.3	1.62 (0.64–4.09)	0.305
Stage (after surgery)							
0–I	48	97.9	1.00		87.5	1.00	
II	66	83.2	7.18 (0.91–56.09)	0.060	86.3	1.18 (0.40–3.48)	0.759
III	27	59.2	20.29 (2.60–58.52)	**0.004**	71.1	2.68 (0.89–8.06)	0.080

Bold values indicate significant *p* values

*HR* hazard ratio, *CI* confidence interval, *HER-2* human epidermal growth factor receptor 2, *BCS* breast conservative surgery, *RT* radiotherapy

^a^Unadjusted Kaplan–Meier estimates

At multivariate analyses, achievement of BCS (*p* = 0.009), tumor grade G1 (*p* = 0.01), and a residual tumor size ≤2 cm after surgery (*p* = 0.005) resulted as independent prognostic factors for DFS, while BCS and grade G1 maintained their significativity for OS (*p* = 0.002 and *p* = 0.005, respectively) [Table 4].

**Table 4:** Multivariate analysis of factors predictive of 5-year disease-free survival and overall survival

	HR (95 % CI)	*p* Value
Disease-free survival		
Grade (G2–G3 vs. G1)	4.56 (1.44–12.42)	**0.010**
Type of surgery (mastectomy vs. BCS)	8.11 (1.68–19.08)	**0.009**
T stage (yT2–yT3 vs. yT0–yT1)	7.92 (1.88–13.43)	**0.005**
Overall survival		
Grade (G2–G3 vs. G1)	4.26 (1.57–11.61)	**0.005**
Type of surgery (mastectomy vs. BCS)	8.86 (2.29–7.47)	**0.002**

Bold values indicate significant *p* values

*BCS* breast conservative surgery

## Discussion

This retrospective study was carried out in postmenopausal breast cancer patients who were candidates for mastectomy with the aim of evaluating the efficacy of NET with AIs in terms of clinical outcome and obtainment of breast conservation.

All women evaluated in the present study received NET with a third-generation AI—letrozole, anastrozole, or exemestane. The three agents are considered equivalent in their antitumor activity and are supposed to have similar efficacy in both the neoadjuvant and adjuvant setting.^23^ BCS was performed in 121 (84 %) of 144 patients. This BCS rate is greater than that reported in clinical studies in which AIs had been administered as the primary treatment in patients with breast cancer.^16^,^17^,^19^ In particular, the P024 study^17^ comparing letrozole versus tamoxifen, the IMPACT^19^ and the PROACT^16^ trials, both comparing anastrozole versus tamoxifen, reported BCS rates of 45, 46, and 43 %, respectively, in the arm receiving AIs. The short duration of NET (only 3 months in the IMPACT and PROACT trials, and 4 months in the P024 study) is likely responsible for the low response rate observed in these studies. In another phase II study, the American College of Surgeons Oncology Group (ACOSOG) Z1031 trial, comparing response to the three AIs in the neoadjuvant setting, the endocrine agents were administered for about 4 months and the overall BCS rate was 83.1 % in the women considered ‘marginal for BCS’ at baseline, and around 50 % in the women categorized as ‘only eligible for mastectomy’.^23^ It is now generally accepted that the minimum duration of NET should be at least 4 months, but better results might be obtained with longer extension of time.^24^,^25^ A recent study comparing 4, 8, and 12 months of neoadjuvant letrozole showed that there was a time-dependent increase in overall response rate, which reflected in BCS rate ranging from 80 to 87.5 %.^26^ In our cohort, the duration of NET did not significantly influence the type of surgery, but the median time of AI administration was 6 months, with 85 % of patients receiving NET for more than 5 months. Moreover, our series encompassed highly endocrine-responsive tumors (ER expression ≥60 %). This may contribute to the high rate of BCS we observed, since the probability of achieving a better response has been related to the level of expression of hormonal receptors.^27^ A trial on exemestane as NET set a cutoff ≥50 % of ER-positive cells for patient eligibility.^28^ The above cited Z1031 trial^23^ required ER-positive disease with high ER expression, i.e. Allred score of 6 to 8.^29^ It has been suggested that tumors with both ER and PR positivity in more than 50 % of the cells might be considered highly endocrine responsive, while a positivity in less than 50 % of the cells predicts an incomplete endocrine responsiveness.^3^ In our study, all patients had tumors with ER expression ≥60 %, and more than half (63 %) had both ER and PR ≥ 50 %.

The variables statistically associated with the achievement of BCS were tumor size <3 cm at diagnosis and HER-2 negativity. The latter result probably reflects the intrinsic hormonal resistance of HER-2-positive tumors.^30^,^31^

BCS was performed in patients reporting clinical objective response (CR + PR) and was significantly associated with longer DFS and OS. Given that achievement of BCS strongly correlates with the clinical response of the primary tumor, the latter was not included as a variable in the multivariate analyses. We observed only two pCRs (1.4 %), in accordance with the low rate reported in NET studies, ranging from 1 to 10 %.^19^,^32^–^34^ pCR, which is the most important prognostic factor for neoadjuvant chemotherapy, is not an appropriate surrogate marker for prognosis in patients with HR-positive tumors. In fact, patients with this subtype of breast cancer rarely obtain a pCR but have a good outcome, even in presence of residual disease.^3^–^8^

Interestingly, our data indicate that grade G1 is a tumor biological characteristic strongly associated with longer DFS and OS. It has long been established that patients with well-differentiated breast cancer, including those with HR-positive tumor, have a better survival than those with G2 and G3 tumors.^35^ However, in the last St. Gallen Consensus Conference,^24^,^36^ the Ki-67 proliferation marker,^37^ rather than grading, was taken into consideration for the separation of HR-positive/HER-2-negative tumors in luminal A and luminal B subtypes, two groups with different prognosis.^38^ This recommendation was based on data suggesting that Ki-67 levels >14 % were able to identify a high-risk group in terms of prognosis.^39^,^40^ In the absence of Ki-67 determination, grading is still used to differentiate luminal molecular subtypes.^8^ The prognostic role of Ki-67 in breast cancer is controversial. Two different meta-analyses of studies on early breast cancer 41,42 and a recent large retrospective study 43 have shown that high levels of Ki-67 are associated with a worse prognosis. In addition, in HR-positive breast cancer, reduction of Ki-67 after 2 weeks of NET correlated with better response and recurrence-free survival.^44^ On the contrary, another study conducted on patients with breast cancer receiving neoadjuvant chemotherapy showed no predictive or prognostic value of Ki-67 in HR-positive/HER2-negative tumors.^45^ Given the great heterogeneity of patients in the different studies, and the different methods utilized to determine and score Ki-67, the American Society of Clinical Oncology (ASCO) Tumor Marker Guidelines Committee did not advise the routine use of Ki-67 for the estimation of prognosis in patients with breast cancer.^46^ In our study, neither baseline expression of Ki-67 nor Ki-67 variations after NET influenced the long-term outcome (data not shown). It has been reported that the prognostic value of Ki-67 is mainly attributed to the histological grade G 2,47,48 and the prevalence of patients with G1 tumors (67 %) in our cohort may justify the lack of prognostic value of Ki-67. Another study evaluated the long-term outcome of patients after NET, showing that a low-risk profile (i.e. pT0/1, pN0, Ki6-67 <2.7 % and Allred score 3–8 for ER status, the so-called PEPI score) allowed to identify a group of patients with a very low risk of disease progression.^49^ Our data are in agreement with these results only for pathological stage, but not for Ki-67 and ER status, likely for the very high ER positivity and G1 tumor percentage in our patients’ population, as emphasized above.

Finally, the high rate of BCS in our study is especially relevant considering that more than 90 % of the patients were older than 65 years. In fact, in elderly patients most authors recommend a conservative surgery based on the increased risk of death from concomitant disease and the reduced risk of local recurrence.^33^,^50^,^51^ Moreover, the preservation of body image in older patients is an important psychological factor influencing both the quality of life and mental health.^52^–^54^

The limits of this retrospective study are mainly represented by single surgery team evaluation for patient eligibility to BCS prior to neoadjuvant therapy and the relatively small number of events to investigate long-term outcomes.

## Conclusions

We show that elderly breast cancer patients with a tumor size <3 cm at diagnosis and HER-2-negativity have a higher probability of achieving BCS after NET. Moreover, patients treated with BCS and with grade G1 tumor have a reduced risk of recurrence and death in the long-term follow-up. It is likely these patients are those who will benefit the most from preoperative endocrine therapy.
